# Hypercoagulopathy in Overweight and Obese COVID-19 Patients: a Single-Center Case Series

**DOI:** 10.2478/jccm-2021-0032

**Published:** 2021-11-13

**Authors:** Azza Sarfraz, Zouina Sarfraz, Aman Siddiqui, Ali Totonchian, Syed Hashim Abbas Ali Bokhari, Hafiza Hussain, Muzna Sarfraz, Gaurav Patel, Muhammad Hassaan Amjad, Sameer Saleem Tebha, Ivan Cherrez-Ojeda, Patrick Dreyer, Harshad Amin, Jack Michel

**Affiliations:** 1 Larkin Health System, South Miami, FL, USA; 2 Pediatrics and Child Health, The Aga Khan University, Karachi, Pakistan; 3 Research and Publications, Fatima Jinnah Medical University, Lahore, Pakistan; 4 Internal Medicine, Dow University of Health Sciences, Karachi, Pakistan; 5 School of Medicine, The Aga Khan University, Karachi, Pakistan; 6 Neurosurgery, Sir Ganga Ram Hospital, Lahore, Pakistan; 7 Allergy and Pulmonology, Universidad de Especialidades Espíritu Santo, Guayaquil, Ecuador; 8 Respiralab Research Center, Guayaquil, Ecuador

**Keywords:** Coronavirus disease 2019, venous thromboembolism, D-dimer, heparin, obesity, disseminated intravascular coagulation, covid-19-associated coagulopathy

## Abstract

A case series is presented of five overweight or obese patients with confirmed coronavirus disease 2019 (COVID-19) in South Miami, Florida, United States. A multitude of coagulation parameters was suggestive of a hypercoagulable state among the hospitalized COVID-19 patients. This article reports various manifestations of hypercoagulable states in overweight and obese patients, such as overt bleeding consistent with disseminated intravascular coagulation, venous thromboembolism, gastrointestinal bleeding as well as retroperitoneal hematoma. All of the required admission to the intensive care unit and subsequently patients died. The characteristics of COVID-19-associated coagulopathy are atypical and warrant a further understanding of the pathophysiology to improve clinical outcomes, specifically in overweight or obese patients.

## Introduction

As of August 9, 2021, coronavirus disease (COVID-19) has infected 203 million individuals and claimed 4.3 million lives worldwide. The pathologic mechanisms that contribute to the severity of COVID-19 and worsening prognosis among overweight and obese patients have been studied [1]. On May 27, 2020, the International Society on Thrombosis and Haemostasis identified intrinsic and extrinsic risk factors for developing COVID-19 associated venous thromboembolism in hospitalized patients comprising of obesity, elevated D-dimer levels (>6 times ULN), c-reactive protein and other indicators of disseminated intravascular coagulation [2]. Thrombotic complications were observed in 14-49% of COVID-19 patients requiring hospitalization despite prophylactic anticoagulation [3,4]. It is challenging to diagnose pulmonary embolism and deep venous thrombosis due to the overlap with COVID-19 symptoms such as shortness of breath, and preliminary guidelines suggest risk-stratified prophylactic and therapeutic anticoagulant use [2,5]. Earlier studies from China reported elevated D-dimers, a fibrin degradation product indicating thrombosis, in 46 to 63% of patients along with thrombocytopenia and prolonged prothrombin time associated with worse clinical outcomes [6,7]. D-dimer is observed to be a useful prognostic marker of disease severity and mortality at admission and during the hospital stay in COVID-19, with levels higher than 1000 ng/mL associated with an 18-fold higher risk of mortality, likely reflective of the coagulative activation from excessive inflammation, platelet activation, endothelial dysfunction, and stasis [7]. The hemodynamic and respiratory effects of COVID-19 have alerted scientific communities across the world [8].

A multitude of coagulation parameters suggestive of a hypercoagulable state has been reported among hospitalized COVID-19 patients.

Each case presentation includes tabulated indicators of clinical, radiological, laboratory, and other imminent findings.

This paper reports the baseline characteristics, clinical features, and outcomes of five overweight or obese COVID-19 patients who died while in hospital.

## Methodology

This single-center, retrospective case series was conducted at Larkin Community Hospital, South Miami, Florida, United States, the only hospital in Miami-Dade County with an area of critical need designation by the Florida Board of Medicine.

The case notes of patients confirmed as COVID-19 positive and hospitalized from May 17 2020, to July 5, 2020, were retrospectively analyzed.

The U.S. Food and Drug Administration (FDA) authorized COVID-19 testing kits had been used for diagnostic testing of SARS-CoV-2 in all patients, as stated in the case reports.

Qualitative detection of nucleic acid from SARS-CoV-2 from the upper respiratory tract, either naso-pharyngeal or oropharyngeal, had been conducted by obtaining sputum swabs, as stated in the case reports.

SARS-CoV-2 had been detected by performing repeated PCR tests on specimens of either pharyngeal or sputum swabs or both, according to laboratory guidance by the World Health Organization, as stated in the case reports.

The patients were diagnosed as stated in the case reports with COVID-19 if they had:

- Any clinical symptoms
- If a positive PCR result for SARS-CoV-2 was obtained.

## Case Presentation

The baseline and clinical characteristics of the five patients during their hospital stay are presented in Table 1.

**Table 1:** Demographic, baseline, and laboratory characteristics of patients at hospital admission.

	Patient 1	Patient 2	Patient 3	Patient 4	Patient 5
**Demographic and baseline characteristics**					
Age, years	68	62	41	64	96
Gender	Male	Male	Female	Female	Male
BMI (kg/m^2^)	31.8	28.2	30.18	35.34	28.74
The severity of COVID-19 at admission	Negative	Mild	Moderate	Moderate	Mild
Previous illnesses	HTN, D.M., and CHF	DM	Asthma	HTN, hypothyroid- ism, breast cancer	HTN, COPD, CAD, D.M., HLD

**Anti-thrombotic use at baseline**					
Anticoagulants	No	No	No	No	Yes
Anti-platelet drugs	No	No	No	Yes	No
Time from symptom onset to start of treatment (days)	13 days	2 days	14 days	5 days	1 day
Symptoms	Exertional dyspnoea and orthopnoea	Fever, cough, and fatigue	Shortness of breath and pro- ductive cough	Shortness of breath and tachypnea	Functional decline

**Vital signs on Day 1 of admission**					
Temperature (°F)	98.7	102.4 (fever)	98.6	98.8	97.9
Respiratory rate (breaths per min)	17	20	24	18	20
Oxygen saturation on room air	98%	97%	89%	97%	100%
Blood pressure (mm Hg)	109/77	144/88	96/65	138/66	148/88
Heart rate (beats per min)	95	69	103	76	76

**Laboratory findings on Day 1 of**	**admission**				
White blood cell count (x10^9^ cells per L)	10.95	5.13	6.3	7.98	7.1
Lymphocyte count (10^9^ cells per L)	1.55	1.09	0.6	0.44	0.73
Neutrophil count (10^9^ cells per L)	8.52	3.65	5.4	6.74	5.23
The neutrophil-to-lympho- cyte ratio (NLR)	5.49	3.35	9	15.32	7.16
Haemoglobin (g/L)	9.7	12.5	11.9	13.3	14.9
Platelet count (10^9^ per L)	246 (Mini-	119 (Mini-	301 (Minimum:	212 (Minimum: 82)	238 (Minimum:
	mum: 174)	mum: 68)	41)		203)
Creatinine (mg/dL)	1.7	1.0	0.8	0.7	1.5
C-reactive protein (mg/dL)	3.4 (Peak: 9.8)	2.7 (Peak: 9)	15.2 (Peak: 16	2.3 (Peak: 16)	7.5 (Peak: 10.44)
ESR (mm/hr)	23	20	78	50	13
LDH (U/L)	174 (Peak:	319 (Peak:	406 (Peak:	261 (Peak: 666)	209 (Peak: 340)
	519)	498)	1316.6)		
Ferritin (ng/mL)	214.9 (Peak:	234.9 (Peak:	338.1 (Peak:	183.2 (Peak: 218.2)	60 (Peak: 123.5)
	455.9)	1506.5)	1506.5)		
D-dimer (ng/mL)	268 (Peak:	367 (Peak:	2506 (Peak:	306 (Peak: 878)	708 (Peak: 3480)
	4201)	4081)	5199)		
Procalcitonin (ng/mL)	0.2	NA	0.2	0.61	0.26

NA: Not available or not applicable, HTN: hypertension, CAD: coronary artery disease, COPD: chronic obstructive pulmonary disease, D.M.: diabetes mellitus, HLD: hepatic liver disease.

### Patient 1

Patient 1 was admitted on June 9, 2020, due to an exacerbation of congestive heart failure. He was tested five times for COVID-19 to assess the concurrent multifocal pneumonia on May 5, May 29, June 10, June 13, and June 21. Each test was negative. A diagnosis of COVID-19 was made on June 25, 2020_,_ following a positive test.

The patient had a BMI of 31.8 kg/m^2^, with comorbidities that included hypertension and diabetes mellitus. On presentation, the complaints were suggestive of exacerbation of congestive heart failure with symptoms limited to orthopnoea and exertional dyspnoea.

He was managed for heart failure with reduced ejection fraction (HFrEF); an echocardiography demonstrated an ejection fraction of 35-40%, requiring thoracentesis and 1000cc removal of pleural fluid.

The procedure was followed by a sharp drop in serum haemoglobin levels, resulting in haemodynamic compromise requiring multiple blood transfusions.

The following medications were given during his hospital stay:

- Azithromycin as Zithromax Injection, (Pfizer^®^, Kalamazoo, Michigan, United States) 500 mg intravenously, was administered from June 9, 2020, until June 11, 2020, on Days 1-3 post-admission.
- Vasopressors as Dobutamine hydrochloride (Pfizer^®^, Kalamazoo, Michigan, United States) were administered from June 10, 2020, until June 28, 2020, on Days 2-19 post-admission, 1 mcg/kg/ min as an intravenous continuous infusion, and 10 mcg/kg/min infusion rate.
- Clopidogrel Bisulfate, as Plavix^®^; (Sanofi, Kansas City, Missouri, United States) was administered from Days 2-19 post-admission, 75 mg, orally once daily. No loading dose was administered.
- Heparin sodium by injection (Pfizer^®^, Kalamazoo, Michigan, United States) as a 5000 units intravenous bolus on June 24, 2020, and then as a continuous intravenous infusion of 1300 UI/hr over Days 15-19 post-admission.

On Day 3, post-admission, multifocal pneumonia was recorded following a computerized tomography scan despite five previous negative tests for COVID-19.

On Day 16, post-admission, the patient was diagnosed with gastrointestinal bleeding following a gastrointestinal bleeding scan.

The patient tested positive for COVID-19 on the same day, and while an order was placed for remdesivir and convalescent plasma, due to the patients deteriorating clinical status and ultimate demise on Day 19 post-admission, they were not be administered.

On Day 18, post-admission, esophagogastroduodenoscopy did not reveal any source of bleeding, and colonoscopy was scheduled for Day 20 post-admission to assess for an occult source. In the late hours of Day 19, the patient’s oxygen started desaturating, measured at 66%, with associated tachypnea and usage of secondary muscles of respiration. The patient was placed on a venti-mask at 50% FiO2 but continued to suffer from dyspnoea, associated with leucocytosis (WBC elevated from 7.9-13 x 10^9^ cells per L since morning).

The patient was upgraded to a 15L non-breathing oxygen mask and was connected to a bilevel positive airway pressure (BiPAP) ventilator. The electrocardiogram (EKG) demonstrated sinus tachycardia and a bilateral ultrasound lower extremity Venous Duplex ultrasound was requested and scheduled for Day 20. However, on Day 19, post-admission, the patient had worsening shortness of breath, indicating respiratory distress. Following thirty-one minutes of cardiopulmonary resuscitation following the advanced cardiac life support protocol, the patient was pronounced dead

### Patient 2

**Fig. 1:**
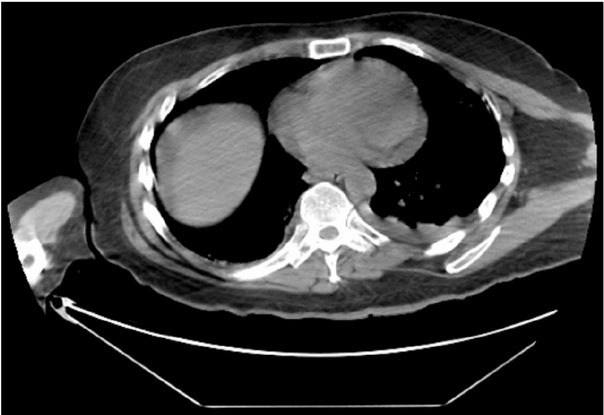
C.T. Scan: Abdomen and Pelvis without contrast on Day 21 of admission.

Patient 2 was admitted on May 20, 2020, after testing positive for COVID-19 on May 19, 2020, with the mild disease at presentation. The BMI of the patient was 28.2 kg/m^2^, with diabetes mellitus as a comorbidity. He complained of fever for the last two days associated with a non-productive cough and lethargy. The patient was managed according to U.S. COVID-19 treatment protocols. He developed a left retroperitoneal hematoma on Day 21 post-admission (Figure 1). A laboratory workup identified worsening anaemia requiring multiple blood transfusions.

The patient was given the following medications:

- Heparin Sodium by injection (Pfizer^®^, Kalamazoo, Michigan, United States) as a 5000 units intravenous bolus on June 5, 2020, and then as a continuous infusion of 1300 UI/hr over Days 16 – 23 post-admission.
- Intravenous remdesivir (Veklury®, Gilead Sciences, La Verne, California, United States) was administered as a loading dose of 200 mg on Day 25 and 100 mg from Days 26-30, post-admission.
- Epinephrine (Pfizer^®^, Kalamazoo, Michigan, United States) 1:1000) solution for injection, 1mg/ml intravenously on Day 47 and Day 52 post-admission.

The patient developed worsening anaemia and respiratory failure, warranting admission to the intensive care unit on Day 24 post-admission. However, the patient’s condition worsened, and he was pronounced dead on Day 52 post-admission.

### Patient 3

Patient 3 was admitted on May 17, 2020, after a 2-week history of shortness of breath with moderate disease at presentation. Her BMI was 30.18 kg/m^2^, with a history of asthma. She had noticed shortness of breath without relief from an albuterol inhaler for two weeks at home. She was admitted to the intensive care unit due to progressive shortening of breath and oxygen desaturation (85%). She was placed on a venti-mask at 50% FiO2 but continued to desaturate, requiring an upgrade to a nonrebreather mask at 100% FiO2 on day 3. On Day 5, the patient developed acute respiratory distress and was switched to BiPAP. Despite having received five doses of intravenous remdesivir (Veklury®, Gilead Sciences, La Verne, California, United States) once daily with a loading dose of 200 mg on Day 3 post-admission, and 100 mg from Days 4-7 post-admission, intravenous tocilizumab (Actemra®, Roche, Oceanside, California, United States) 800 mg on Day 4 post-admission with an additional intravenous infusion on Day 5 post-admission, and convalescent plasma on Day 8 post-admission, the patient continued to deteriorate. On

Day15 post-admission, subcutaneous emphysema and pneumomediastinum were noted on the C.T. scan. The patient improved over the next few days and received extended-spectrum antibiotics to treat the development of sepsis. On Day 16 post-admission, she developed mucosal bleeding accompanied by thrombocytopenia with a recorded platelet count of 41. This raised the suspicion of heparin-induced thrombocytopenia Type II. Heparin-induced thrombocytopenia was not suggested on diagnostic testing, and disseminated intravascular coagulation was suspected following sepsis. The following prophylactic anti-thrombotic therapy was continued; oral clopidogrel bisulfate ( PLAVIX^®,^ Sanofi, Kansas City, Missouri, United States) 75 mg once daily, without a loading dose. The medication continued to be administered from Day 16 post-admission until the end of her hospital stay.

On Day 33, a right-sided pneumothorax was observed, and the patient’s condition worsened. Additionally, the patient continued to desaturate with high oxygen demands and persistent tachycardia (R.R.>30 per minute). During the hospital stay, the patient was administered heparin sodium (Pfizer^®^, Kalamazoo, Michigan, United States), a 5000 units intravenous bolus on Day 13 post-admission, and then a continuous intravenous infusion of 1300 UI/hr until Day 21 post-admission. Additionally, an intravenous vasopressin injection of Vasostrict® (Par Pharmaceutical, Rochester, Michigan, United States) 1mL, 20U, on Day 39 post-admission.

She was pronounced dead on Day 39 post-admission.

### Patient 4

The patient presented on with complaints of shortness of breath and tachypnea for the previous five days pre-admission. She tested COVID-19 PCR positive on Day 1post-admission, consistent with moderate illness. The patient had a BMI of 35.34 kg/m2 and comorbidities of hypertension, hypothyroidism, and breast cancer. She had episodes of hypoxia requiring BiPAP to maintain her oxygen saturation. The patient received six doses of remdesivir from Day 2 and tocilizumab on Day 6 post-admission.

Heparin Sodium by injection (Pfizer^®^, Kalamazoo, Michigan, United States) as a 5000 units intravenous bolus on Day 6 post-admission, and then as a continuous intravenous infusion of 1300 UI/hr until Day 29 post-admission.

From Days 30-37 post-admission, she was hypotensive and was managed on an intravenous vasopressin injection of Vasostrict® (Par Pharmaceutical, Rochester, Michigan, United States) Sol: 1mL, 20U, and intravenous epinephrine (Pfizer^®^, Kalamazoo, Michigan, United States) 1mg/ml IV (1:1000).

On Day 31, the patient was hypoxic and tachypneic with a respiratory rate >30 per minute and was consequently intubated and mechanically ventilated. However, on Day 32, blood cultures were positive for *staphylococcus epidermidis* and treated with appropriate antibiotics. On day 37, a subsegmental pulmonary embolism was noted on the C.T. scan despite having received a prophylactic anti-thrombotic administration of 75 mg, orally, once daily, of clopidogrel bisulfate (PLAVIX^®^; (Sanofi, Kansas City, Missouri, United States) No loading dose had been given. This medication was administered throughout the hospital stay.

The patient was in respiratory distress, and life-saving cardiopulmonary measures were carried out. Unfortunately, she was pronounced dead after seventeen minutes of cardiopulmonary resuscitation on Day 37.

### Patient 5

The patient was admitted with complaints of functional decline and had been recently diagnosed with acute kidney injury. In addition, the patient had a BMI of 28.74 kg/m^2^ and a history of hypertension, chronic pulmonary obstructive disease, coronary artery disease, diabetes mellitus, and hepatic liver disease.

After testing PCR-positive for COVID-19 on Day 1 post-admission and symptoms consistent with mild disease, treatment for COVID-19 was initiated. On Day 3, a C.T. scan revealed multifocal interstitial pneumonia present on repeat chest x-rays. He was receiving prophylactic anti-thrombotic therapy. The patient developed hypokalaemia during his stay, which was corrected,

The following medications were administered:

- Heparin Sodium by injection (Pfizer^®^, Kalamazoo, Michigan, United States) as a 5000 units intravenous bolus on Day 1 post-admission, and then as a continuous intravenous infusion of 1300 UI/hr until Day 17 post-admission.
- Clopidogrel Bisulfate, as Plavix^®^; (Sanofi, Kansas City, Missouri, United States) was administered from Days 2-11 post-admission, as 75 mg, orally once daily.

Due to the decline in the patient’s health, 200mg of remdesivir (Veklury®, Gilead Sciences, La Verne, California, United States) was administered on Day 9, and 100 mg from Days 10-14. On Day 16, a Doppler ultrasound of the right upper extremity showed a thrombus in the right axillary vein. Consequently, the patient developed leucocytosis (WBC 22.8 x 10^9^ cells per L) and sepsis. Appropriate protocol for sepsis was initiated, but the patient started declining and was declared dead on the night of Day 16.

### Further Notes

**Fig. 2:**
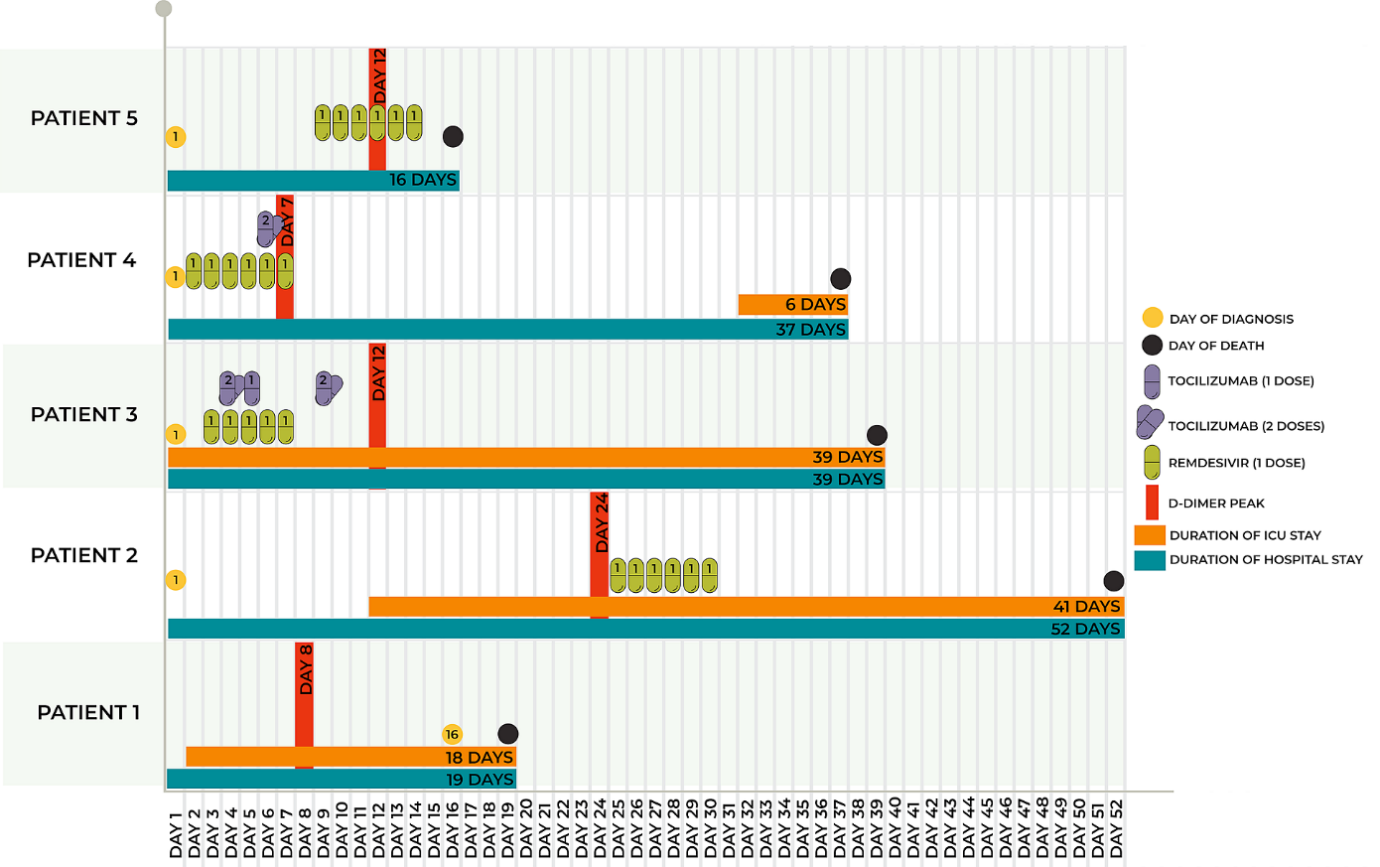
Significant events of all patients during hospital stay from day 1 of admission.

Schematic representations of the major events of each of the patients during the hospital stay are summarized for each patient day by day from Day 1 post-admission in Figure 2.

All patients were diagnosed with COVID-19 on Day 1 post-admission except Patient 1. Various manifestations of hypercoagulable states were reported , possibly linked to medication use and infection with COVID-19, in overweight and obese patients, such as overt bleeding consistent with disseminated intravascular coagulation, venous thromboembolism, G.I. bleeding as well are retroperitoneal hematoma.

All the patients required intensive care on admission and subsequently died (Table 1).

## Discussion

The findings highlight key issues to address in the management of COVID-19 patients. First, close monitoring of obese COVID-19 patients, especially those warranting critical care, is required to prevent complications of hypercoagulability. Obesity is a known prothrombotic state due to its activation of chronic inflammation and impaired fibrinolysis. Specifically, effector mechanisms of obesity include endothelial dysfunction, plaque rupture with exposure of tissue factors, activation of platelets, and delayed lysis of clots [9]. Nearly 48% of obese adult COVID-19 patients were hospitalized across fourteen states in the United States in March 2020 [10]. Secondly, consistent with previous cohorts, the current series of patients developed haemostatic abnormalities, including mild thrombocytopenia and elevated D-dimer levels requiring admission to the intensive care unit.

Along with the underlying stasis, coagulopathy observed in COVID-19 predisposes to thrombotic events, observed in Patients 4 and 5. In a cohort of 183 patients, the International Society on Thrombosis and Haemostasis criteria for probable disseminated intravascular coagulation was met for 71% of patients, and non-survivors had a 3.5-fold increase in D-dimer levels; however, a significant clinical finding of acute decompensated disseminated intravascular coagulation is bleeding, which may not corroborate with the clinical findings in COVID-19 patients [11]. Hyper-coagulable parameters include the prolongation of prothrombin time and partial thromboplastin time and the elevation of fibrinogen, d-dimer, and von Wille-brand factor (vWF) activity without profound thrombocytopenia. The lack of significant thrombocytopenia and no schistocytes seen on a peripheral blood smear is discordant with acute decompensated disseminated intravascular coagulation [12]. Third, the patients in the presented series were overweight, obese, or morbidly obese; incorporating weight-adjusted anticoagulation and appropriate haemostatic therapies is necessary for this subset of patients. Increased efficacy of weight-adjusted heparin prophylaxis in obese hospitalized post-surgery patients has been observed with a 1.5% overall reduction in venous thromboembolism [13]. While therapeutic dosing of unfractionated heparin and low-molecular-weight heparin is not currently endorsed by preliminary guidelines, consideration should be given to performing routine surveillance using venous Doppler ultrasound for obese COVID-19 patients admitted to an intensive care unit as well as to administer a therapeutic dose of anticoagulation to prevent venous thromboembolism [14].

The characteristics of COVID-19-associated coagulopathy are distinct from disseminated intravascular coagulation and sepsis-induced coagulopathy and warrant a further understanding of the pathophysiology to reduce mortality rates in COVID-19 as previously reported in a cohort of 24 critically ill patients [12]. Furthermore, as intermediate- or higher-dose unfractionated heparin or low-molecular-weight heparin are being administered during COVID-19 treatment according to venous thromboembolism risk stratification, e.g. obesity or morbid obesity, patients may be at increased risk to develop heparin-induced thrombocytopenia manifesting as moderate-to-severe thrombocytopenia [15]. However, higher platelet counts at baseline may preclude diagnosing heparin induced thrombocytopenia that warrants closer monitoring and anticoagulation be changed to an alternative, e.g., Fondaparinux, as suspected in Patient 3 [16]. Another unique finding is the correlation of elevated platelet counts and laboratory surrogates of inflammation with increased thromboembolic events in COVID-19 [15]. This has led to the postulation of immunothrombosis via angiotensin-converting enzyme-2 (ACE-2) activation of endothelial cells with endothelial dysfunction, complement activation, platelet and leukocyte recruitment, and resultant thrombin generation [18].

In addition, recent gene expression studies have demonstrated that COVID-19 is associated with platelet hyper-reactivity [19]. Challenges to effectively prevent and treat sequelae of CAC in obese COVID-19 patients are due to the likely lack of efficacy of standard anti-thrombotic dosing regimens for prophylaxis and treatment compared to normal-weight patients.

## Conclusion

Given the resemblance of CAC with other life-threatening pathogenesis, including disseminated intravascular coagulation, the understanding of the clinical features is necessary to inform clinical practitioners when providing care to obese COVID-19 patients, especially in ICU settings. In addition, preliminary data suggest the synergistic mechanisms of obesity and severe acute respiratory syndrome coronavirus 2 (SARS-CoV-2) to predisposing these patients to the occurrence of thromboembolic events and higher mortality rates. Future clinical studies must establish the type, dose, and optimal duration of anticoagulation therapy to ascertain the optimal preventive management strategy in hypercoagulability. Further, a correlation with the rate of venous thromboembolism, particularly in obese COVID-19 patients, ought to be made. Finally, given what we know about platelet hyperactivity in COVID-19, additional anti-platelet prophylactic and therapeutic strategies will need to be investigated.
